# Non-linear relationship of gamma-glutamyl transpeptidase to lymphocyte count ratio with the recurrence of hepatocellular carcinoma with staging I–II: a retrospective cohort study

**DOI:** 10.1186/s13027-022-00428-0

**Published:** 2022-04-08

**Authors:** Zeping Li, Lili Liang, Wen Duan, Chengmao Zhou, Jian-Jun Yang

**Affiliations:** Department of Anesthesiology, Pain and Perioperative Medicine, The First Affiliated Hospital of Zhengzhou University, Zhengzhou, 450000 China

**Keywords:** Hepatocellular carcinoma, Gamma-glutamyl transpeptidase to lymphocyte count ratio, Recurrence, No-linear

## Abstract

**Background:**

High recurrence rate was a major factor for the poor postoperative prognosis of hepatocellular carcinoma (HCC) patients. The present study was intended to evaluate the association of gamma-glutamyl transpeptidase to lymphocyte count ratio (GLR) and the recurrence of HCC with staging I–II in Chinese.

**Methods:**

The retrospective cohort data was derived from the First Affiliated Hospital of Zhengzhou University from January 2014 to December 2018 on 496 patients who underwent radical resection of HCC with staging I–II. Multivariable Cox regression models were used to determine hazard ratios (HR) and 95% confidence interval (CI) for the recurrence of HCC with staging I–II of each GLR tertile category. The restricted cubic spline model was used to find out the threshold effect.

**Results:**

With the low tertile of GLR as the reference, multivariable-adjusted HR and 95% CI of the middle and high tertile categories were 1.748 (1.170–2.612) and 2.078 (1.339–3.227). In addition, there was a positive correlation (HR 1.002; 95% CI 1.001–1.004) and a non-liner relationship was found, whose point was 27.5. When the GLR was less than 27.5, the risk of recurrence increased, obviously with the increase in GLR levels (HR 1.041; 95% CI 1.014–1.068).

**Conclusions:**

The GLR was independently associated with the recurrence of HCC patients with staging I–II. Furthermore, the relationship was positive and no-linear.

## Introduction

Hepatocellular carcinoma (HCC) accounted for 75%-85% of primary liver cancer and was one of the leading causes of death related to tumor in the world [1]. In China, it accounted for 46.7% of global cases of liver cancer in 2018 [2]. Compared with HCC patients with staging III-IV (American Joint Committee on Cancer pathological TNM stage, 7^th^), there were more choices for treatments for HCC patients with staging I–II. Surgical resection may be the first therapeutic option for HCC patients in stages I–II, who have a better prognosis after surgery than HCC patients in stages III-IV [3, 4]. Despite advancements in HCC early detection, surgical technology, and postoperative monitoring, the overall prognosis of HCC patients after radical resection has remained unsatisfactory due to the high postoperative recurrence rate [5]. Therefore, it was vital to adequately understand the risk factors for the recurrence of HCC patients with staging I–II, which can be used to guide clinical decision making and improve patient prognosis.

Inflammatory and immune factors both played an important role in carcinogenesis and tumor recurrence [6, 7]. Meanwhile, routine blood tests and liver function were essential for peripheral blood examinations of HCC patients, so it was convenient to acquire the indicators of liver function, immunity and inflammation, such as liver enzymes and lymphocytes. Liver function indicators could reflect the liver's state directly, and were associated with HCC patients' prognosis. Recently, several studies reported that the aspartate aminotransferase to neutrophil ratio index (ANRI), aspartate aminotransferase to platelet ratio index (APRI), platelet to albumin ratio (PAR) and neutrophil times γ-glutamyl transpeptidase to lymphocyte ratio (NγLR) were independent effective predictors of the prognosis of patients with HCC [8–11]. Similarly, the preoperative gamma-glutamyl transferase to lymphocyte ratio (GLR) has been found to have a significant prognostic value in AFP-negative HCC patients with a single tumor size ≤ 5 cm undergoing resection [12–14]. However, the prognostic value of GLR has not been reported in the monitoring of the recurrence of HCC patients with staging I–II following curative resection.

Therefore, the purpose of the retrospective cohort study was to evaluate the association between GLR and the recurrence of HCC patients with staging I–II and to explore their dose–response relationship, which may guide the development of postoperative therapeutic approaches.

## Materials and methods

### Study population

The study included 496 HCC patients who underwent radical resection at the First Affiliated Hospital of Zhengzhou University, from January, 2014 to December, 2018. The retrospective study was approved by the Medical Ethics Committee of the First Affiliated Hospital of Zhengzhou University. The data was anonymous, and the need for informed consent was therefore waived. The patients who met the following criteria were included in this study: (1) The postoperative pathological result was hepatocellular carcinoma; (2) patients ≥ 18 years old with initial radical hepatectomy; and (3) with staging I–II. The patients who met the following criteria were ruled out: (1) HCC complicated by other fatal diseases or primary tumors; (2) Patients who received preoperative chemotherapy and/or radiotherapy; (3) Patients who had a disease of the immune system or blood system.

### Follow up and data extraction

The follow-up time ended in July, 2019, lost to follow-up or death. Follow-up was conducted every three months after surgery. The postoperative patients who did not come to our hospital for reexamination were followed up by telephone. The baseline information included age, sex, smoking, drinking, Child–Pugh class, HCC family history, operation time, differentiation, microvascular invasion, ASA (American Society of Anesthesiologists physical status classification system), urine volume, blooding volume, allogeneic blood, Hemoglobin (HB), platelet (PLT), lymphocyte count, gamma-glutamyl transpeptidase (GGT), alanine aminotransferase (ALT), aspartate aminotransferase (AST), alkaline phosphatase (ALP), hepatitis B surface antigen (HBsAg), tumor number; tumor-node-metastasis (TNM), tumor size, alpha-fetoprotein (AFP), and recurrence. Recurrence was defined as the presence of clinical symptoms correlated with hepatic ultrasound, computed tomography (CT) or serum alpha-fetoprotein abnormal conditions.

### Statistical analysis

The primary aim of the analysis was to explore the association between GLR and the recurrence of HCC with staging I–II. To examine the relationship between GLR and the recurrence of HCC with staging I–II, we performed three different models using the univariate and multivariate Cox proportional-hazards regression model, including the non-adjusted model (no covariates were adjusted), adjusted model I (adjusted for age and sex) and adjusted model II (covariates were included as potential confounder in model II if they changed the estimates of GLR on recurrence by more than 10% or were significantly associated with recurrence). Effect sizes with 95% confidence intervals were recorded. For baseline characteristics analysis, the chi-square tested for categorical variables, the one-way ANOVA tested for continuous variables, or the Kruskal-Whallis tested for skewed distribution were used for the evaluation of the differences among the tertiles of GLR. Data were expressed as mean ± standard deviation (SD) (Gaussian distribution) or median (min, max) (Skewed distribution) for continuous variables, and frequency or percentage for categorical variables.

To account for the non-linear correlation between GLR and the recurrence of HCC with staging I–II, the Cox proportional hazards regression model with cubic spline functions and the smooth curve fitting (penalized spline method) were used to address nonlinearity. In addition, a two-piece-wise Cox proportional-hazards regression model was used to find the threshold effect. The recurrence-free survival (RFS) according to the threshold of GLR on postoperative RFS in different follow-up time was depicted and analyzed with the Kaplan–Meier survival analysis with the log-rank test.

We performed stratified analyses to explore potential indicators that may modify the relationship between GLR and the recurrence of HCC with staging I–II. The modification across subgroups was inspected by the likelihood ratio test. Finally, we converted GLR into a categorical variable according to the tertiles of GLR, and calculated the *P* for trend in order to confirm the results of GLR as a continuous variable, and to examine the nonlinearity.

Data analyses used R (http://www.R-project.org) and Empower(R) (www.empowerstats.com, X&Y solutions, Inc., Boston, MA). *P* values < 0.05 (two-sided) were considered statistically significant.

## Results

### Subject characteristics

Characteristics of the study patients stratified by GLR tertiles were displayed in Table 1. Of 496 HCC patients included in the final analyses, 199 (40.121%) patients developed recurrences. A total of 165 patients were in the low GLR group (tertile 1: GLR < 20.442), 165 patients were in the middle GLR group (tertile 2: 20.442–50.382), and 166 patients were in the high-GLR group (tertile 3: GLR ≥ 50.382). The subjects included 102 women and 394 men. Patients with high GLR values (GLR ≥ 50.382) were more likely to receive allogeneic blood therapy and to report a history of smoking and drinking; they also had higher levels of ALT、AST、ALP, operation time, blooding volume, tumor size, and recurrence, but lower PLT.

**Table 1:** Baseline characteristics of the study patients according to the tertiles of GLR (n = 496)

Variables	Tertile 1 (< 20.442, n = 165)	Tertile 2 (20.442–50.382, n = 165)	Tertile 3 (> 50.382, n = 166)	*P*-value
Age (years)				0.133
≤ 60	111 (67.273%)	113 (68.485%)	127 (76.506%)	
> 60	54 (32.727%)	52 (31.515%)	39 (23.494%)	
Sex				0.003
Male	117 (70.909%)	135 (81.818%)	142 (85.542%)	
Female	48 (29.091%)	30 (18.182%)	24 (14.458%)	
Smoking				< 0.001
No	123 (74.545%)	102 (61.818%)	91 (54.819%)	
Yes	42 (25.455%)	63 (38.182%)	75 (45.181%)	
Drinking				0.015
NO	140 (84.848%)	128 (77.576%)	119 (71.687%)	
Yes	25 (15.152%)	37 (22.424%)	47 (28.313%)	
HCC family history				0.666
No	142 (86.061%)	147 (89.091%)	147 (88.554%)	
Yes	23 (13.939%)	18 (10.909%)	19 (11.446%)	
HBsAg				0.744
Negative	24 (14.545%)	21 (12.727%)	26 (15.663%)	
Positive	139 (84.242%)	139 (84.242%)	137 (82.530%)	
NA	2 (1.212%)	5 (3.030%)	3 (1.807%)	
ALT (U/L)	23.000 (8.000–101.000)	28.000 (10.000–265.000)	38.000 (10.000–605.000)	< 0.001
AST (U/L)	24.000 (12.000–89.000)	29.000 (14.000–159.000)	40.500 (15.000–386.000)	< 0.001
ALP (U/L)	77.058 (22.856)	87.962 (29.539)	116.245 (68.271)	< 0.001
HB (g/L)	134.727 (15.461)	135.273 (17.457)	131.333 (20.427)	0.096
PLT (10^9/L)	153.073 (51.750)	136.170 (57.049)	124.476 (69.775)	< 0.001
AFP (ng/ml)				0.071
≤ 20	70 (44.872%)	82 (51.572%)	62 (38.750%)	
> 20	86 (55.128%)	77 (48.428%)	98 (61.250%)	
Child–Pugh class				0.065
A	162 (98.182%)	156 (94.545%)	154 (92.771%)	
B	3 (1.818%)	9 (5.455%)	12 (7.229%)	
Differentiation				0.615
Well-moderate	144 (91.139%)	145 (90.062%)	137 (87.821%)	
Poor	14 (8.861%)	16 (9.938%)	19 (12.179%)	
Lymphovascular invasion				0.224
No	130 (78.788%)	137 (83.030%)	125 (75.301%)	
Yes	35 (21.212%)	28 (16.970%)	41 (24.699%)	
ASA				0.377
I	19 (11.585%)	13 (7.927%)	19 (11.446%)	
II	137 (83.537%)	135 (82.317%)	136 (81.928%)	
III	8 (4.878%)	16 (9.756%)	11 (6.627%)	
Operation time (min)	140.745 (46.511)	166.097 (58.854)	177.175 (63.115)	< 0.001
Urine volume (ml)	300.00 (0.000–1200.000)	350.000 (20.000–3500.000)	300.000 (50.000–2150.000)	0.069
Bleeding volume (ml)	200.000(5.000–1400.000)	300.000 (20.000–3500.000)	400.000 (20.000–5000.000)	< 0.001
Allogeneic blood				< 0.001
No	156 (95.122%)	146 (89.571%)	122 (73.494%)	
Yes	8 (4.878%)	17 (10.429%)	44 (26.506%)	
Tumor number				0.864
Single	154 (93.333%)	154 (93.333%)	157 (94.578%)	
Multiple	11 (6.667%)	11 (6.667%)	9 (5.422%)	
TNM				0.183
I	122 (73.939%)	128 (77.576%)	114 (68.675%)	
II	43 (26.061%)	37 (22.424%)	52 (31.325%)	
Tumor size (cm)				< 0.001
≤ 5	132 (80.000%)	108 (65.455%)	92 (55.422%)	
> 5	33 (20.000%)	57 (34.545%)	74 (44.578%)	
Recurrence				< 0.001
No	120 (72.727%)	93 (56.364%)	84 (50.602%)	
Yes	45 (27.273%)	72 (43.636%)	82 (49.398%)	

Data presented were mean ± SD, median (Min–Max), or N (%)

ALT, alanine aminotransferase; AST, aspartate aminotransferase; ALP, alkaline phosphatase; Hb, Hemoglobin; PLT, platelet; AFP, alpha-fetoprotein; HBsAg, hepatitis B surface antigen; ASA, American society of Aneshesiologists physical status classification system; TNM, tumor-node-metastasis; GLR, gamma-glutamyl transpeptidase to lymphocyte count ratio

### Association of non-recurrence and recurrence of HCC with staging I–II

In the univariate analysis, sex, smoking, HBsAg, ALT, AST, ALP, GLR, AFP, vascular invasion, blooding volume, allogeneic blood, TNM and tumor size were significantly associated with the recurrence of HCC with staging I–II (Table 2).

**Table 2:** Univariate analysis to identify risk factors associated with the recurrence of HCC patients with staging I–II

Variables	Statistics	HR 95%CI	*P* Value
Age (years)			
≤ 60	351 (70.766%)	1.0	
> 60	145 (29.234%)	0.742 (0.540, 1.020)	0.06634
Sex			
Male	394 (79.435%)	1.0	
Female	102 (20.565%)	0.638 (0.435, 0.937)	0.02180
Smoking			
No	316 (63.710%)	1.0	
Yes	180 (36.290%)	1.346 (1.016, 1.783)	0.03872
Drinking			
No	387 (78.024%)	1.0	
Yes	109 (21.976%)	1.208 (0.878, 1.662)	0.24598
HCC family history			
No	436 (87.903%)	1.0	
Yes	60 (12.097%)	1.248 (0.837, 1.861)	0.27741
HBsAg			
Negative	71 (14.609%)	1.0	
Positive	415 (85.391%)	1.627 (1.024, 2.583)	0.03917
ALT (U/L)	30.00 (8.00–605.00)	1.002 (0.999, 1.004)	0.20455
AST (U/L)	30.00 (12.00–386.00)	1.005 (1.001, 1.008)	0.00610
ALP (U/L)	93.767 ± 47.817	1.006 (1.004, 1.009)	< 0.00001
GLR	30.61 (3.00–636.84)	1.003 (1.002, 1.004)	< 0.00001
HB (g/L)	133.773 ± 17.953	1.011 (1.003, 1.019)	0.01016
PLT (10^9/L)	137.879 ± 61.046	1.001 (0.998, 1.003)	0.62340
AFP (ng/ml)			
≤ 20	214 (45.053%)	1.0	
> 20	261 (54.947%)	1.605 (1.199, 2.150)	0.00149
Child–Pugh class			
A	472 (95.161%)	1.0	
B	24 (4.839%)	1.050 (0.572, 1.930)	0.87432
Differentiation			
Well-moderate	426 (89.684%)	1.0	
Poor	49 (10.316%)	0.932 (0.586, 1.484)	0.76792
Lymphovascular invasion			
No	392 (79.032%)	1.0	
Yes	104 (20.968%)	2.337 (1.705, 3.204)	< 0.00001
ASA			
I	51 (10.324%)	1.0	
II	408 (82.591%)	1.007 (0.638, 1.587)	0.97718
III	35 (7.085%)	0.947 (0.482, 1.864)	0.87588
Operation time (min)	161.371 ± 58.525	1.002 (1.000, 1.005)	0.03542
Urine volume (ml)	300.00(0.00–2150.00)	1.000 (1.000, 1.001)	0.39094
Blooding volume (ml)	300.00 (5.00–5000.00)	1.000 (1.000, 1.000)	0.18443
Allogeneic blood			
No	424 (86.004%)	1.0	
Yes	69 (13.996%)	1.542 (1.070, 2.223)	0.02024
Tumor number			
Single	465 (93.750%)	1.0	
Mutiple	31 (6.250%)	1.885 (1.127, 3.150)	0.01565
TNM			
I	364 (73.387%)	1.0	
II	132 (26.613%)	2.443 (1.813, 3.292)	< 0.00001
Tumor size (cm)			
≤ 5	332 (66.935%)	1.0	
> 5	164 (33.065%)	1.633 (1.229, 2.170)	0.00071

Data presented were mean ± SD, median (Min–Max), or N (%); HR and 95% CI

### Association of GLR and the recurrence of HCC with staging I–II

Table 3 showed that the association between GLR and the risk of recurrence was determined. With adjustment for potential confounders, increased GLR had a positive relationship with the recurrence of HCC with staging I–II (HR 1.002; 95% CI 1.001- 1.004). In the adjusted model II, the hazard ratios (95%CI) for the recurrence of HCC with staging I–II across GLR tertiles were as follows: The low tertile: < 20.442 (1.0), the middle tertile: 20.442–50.382 (1.748 (1.170–2.612)), the high tertile: > 50.382 (2.078 (1.339–3.227)), independent of age, sex, smoking, operation time, lymphovascular invasion, allogeneic blood, HB, AST, ALP, HBsAg, tumor number, tumor size, AFP.

**Table 3:** Association between GLR and the risk of recurrence of HCC with staging I–II

Exposure	Non-adjusted	Adjust I	Adjust II
GLR	1.003 (1.002, 1.004) < 0.00001	1.003 (1.002, 1.004) < 0.00001	1.002 (1.001, 1.004) 0.00884
GLR tertiles			
Low	1.0	1.0	1.0
Middle	1.899 (1.308, 2.758) 0.00075	1.827 (1.254, 2.660) 0.00168	1.748 (1.170, 2.612) 0.00644
High	2.375 (1.646, 3.426) < 0.00001	2.263 (1.565, 3.273) 0.00001	2.078 (1.339, 3.227) 0.00112
*P* for trend	< 0.00001	0.00001	0.00127

Data presented were HRs and 95% CIs. Non-adjusted model adjusted for: None; Adjust I model adjusted for: age; sex; Adjust II model adjusted for: age; sex; smoking; operation time; lymphovascular invasion; allogeneic blood; HB; AST; ALP; HBsAg; Tumor number; Tumor size; AFP

### Threshold effect analysis of GLR on incident recurrence of HCC with staging I–II

**Fig. 1:**
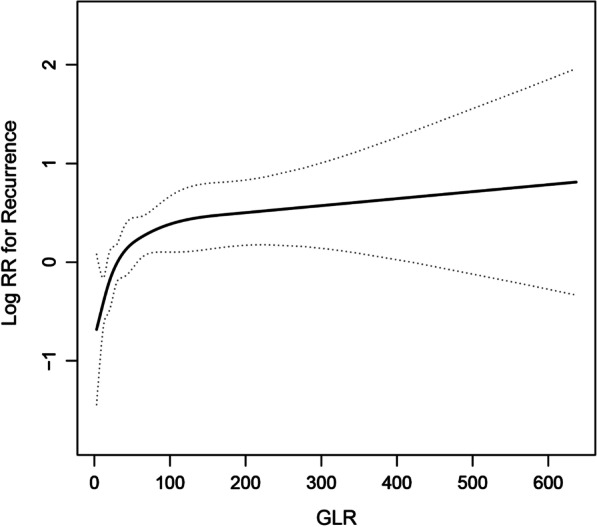
Dose–response relationship between GLR and the risk of recurrence of HCC patients with staging I–II. *Notes* Adjusted for age; sex; smoking; operation time; lymphovascular invasion; allogeneic blood; HB; AST; ALP; HBsAg; tumor number; tumor size; AFP

To confirm whether a dose–response relation between GLR and incident recurrence of HCC with staging I–II existed, we performed a smoothing function analysis. After adjusting for age, sex, smoking, operation time, lymphovascular invasion, allogeneic blood, HB, AST, ALP, HBsAg, tumor number, tumor size and AFP, a nonlinear relationship between GLR and incident recurrence was found (Fig. 1). Using a two-piecewise Cox proportional-hazards regression model, we found that incident recurrence was positively correlated with the GLR until it peaked at 27.5. However, when the GLR was higher than 27.5, the hazard ratio for risk of developing recurrence was 1.001 (95% CI 1.000–1.003), indicating that there was no significant association between the risk of developing recurrence and increased GLR (*P* = 0.0997) (Table 4).

**Table 4:** Threshold effect analysis of GLR on incident recurrence of HCC with staging I–II

Outcome: recurrence	HR (95%IC)	*P* value
One-line linear regression model	1.002 (1.001, 1.004)	0.0088
Two-piecewise linear regression model		
GLR ≤ 27.5	1.041 (1.014, 1.068)	0.0028
GLR > 27.5	1.001 (1.000, 1.003)	0.0997
Log-likelihood ratio test	0.003	

Adjusted for age; sex; smoking; operation time; lymphovascular invasion; allogeneic blood; HB; AST; ALP; HBsAg; tumor number; tumor size; AFP

In Fig. 1, the full line indicated the estimated risk of incident recurrence, and the vacant lines represented a point-wise 95% confidence interval, adjusted for age, sex, smoking, HCC family history, operation time, lymphovascular invasion, allogeneic blood, HB, AST, ALP, HBsAg, tumor number, tumor size, AFP.

### Subgroup analyses

HBsAg (*P* = 0.0341) and AFP (*P* = 0.0416) were interaction factors between GLR and the recurrence of HCC with staging I–II. Limiting the analysis to HBsAg negative patients showed a significant positive relationship between GLR and recurrence (HR 1.007; 95% CI 1.003–1.012; *P* = 0.0023), whereas this relationship was no longer significant in HBsAg positive patients. Similarly, the positive relationship was significant in AFP-negative (≤ 20 ng/ml) patients, but was not significant in AFP-positive (> 20 ng/ml) patients. After adjustment for potential confounding variables, we found that the relationship between GLR and recurrence did not change by age, sex, allogeneic blood, lymphovascular invasion, tumor number, tumor size (all *P* for interaction > 0.05) (Table 5).

**Table 5:** Subgroup analysis of the association between GLR and the risk of recurrence of HCC with staging I–II

Parameters	N		HR	95%CI Low	95%CI High	*P* value	*P* (interaction)
Age (years)							0.4760
≤ 60	329	1.002		1.000	1.004	0.0313	
> 60	133	1.003		1.000	1.007	0.0664	
Sex							0.6469
Male	367	1.002		1.000	1.004	0.0169	
Female	95	1.003		0.998	1.008	0.2005	
Smoking							0.2988
No	298	1.001		0.999	1.004	0.3412	
Yes	164	1.003		1.001	1.005	0.0047	
Allogeneic blood							0.1524
No	396	1.002		1.000	1.003	0.0514	
Yes	66	1.005		1.001	1.010	0.0209	
HBsAg							0.0341
Negative	69	1.007		1.003	1.012	0.0023	
Positive	393	1.002		1.000	1.003	0.0589	
AFP (ng/ml)							
≤ 20	208	1.005		1.002	1.009	0.0014	0.0416
> 20	254	1.002		1.000	1.003	0.1016	
Lymphovascular invasion							0.0980
No	362	1.001		0.999	1.003	0.2805	
Yes	100	1.004		1.001	1.006	0.0011	
Tumor number							0.0654
Single	431	1.002		1.000	1.003	0.0662	
Multiple	31	1.006		1.002	1.009	0.0011	
Tumor size (cm)							0.2891
≤ 5	309	1.003		1.001	1.005	0.0057	
> 5	153	1.001		0.999	1.004	0.2617	

Adjust for age; sex; smoking; operation time; lymphovascular invasion; allogeneic blood; HB; AST; ALP; HBsAg; tumor number; TNM; tumor size; AFP

**Fig. 2:**
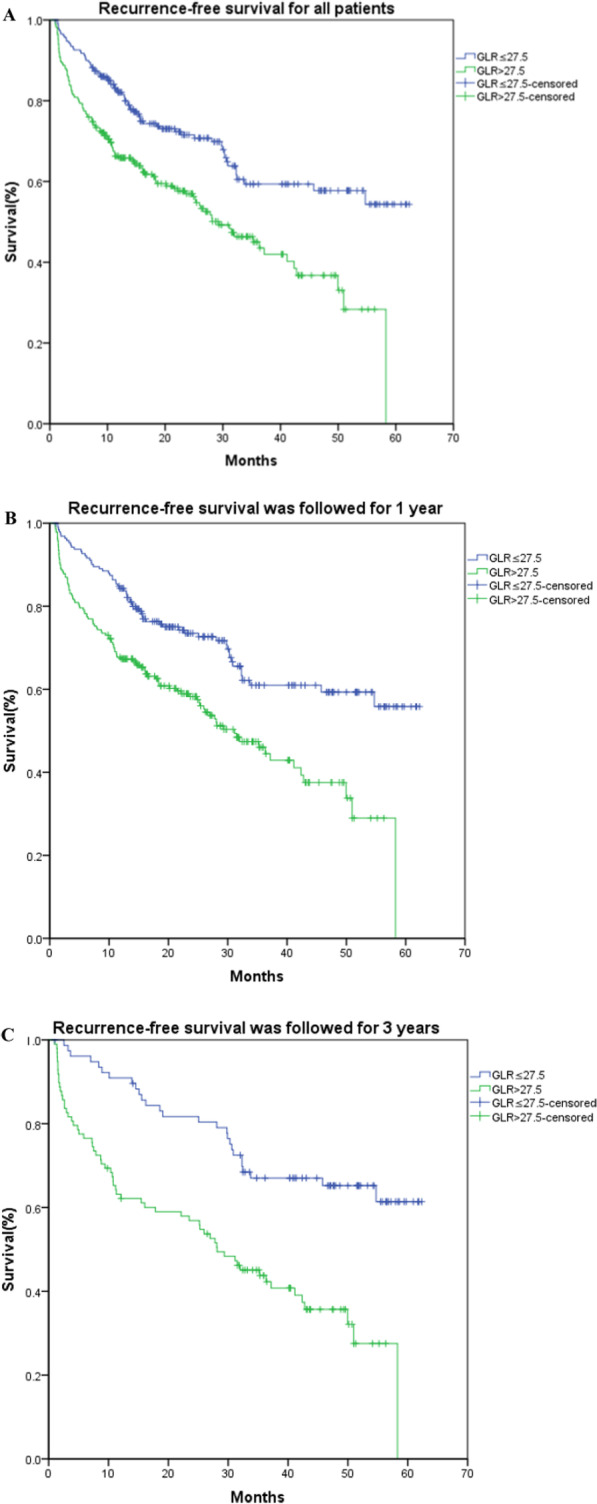
Kaplan–Meier survival curves from the date of surgery by the threshold of GLR. **A** 496 patients from January, 2014 to December, 2018 (*P* < 0.001), **B** 422 patients from January, 2014 to July, 2018 (*P* < 0.001), **C** 176 patients from January, 2014 to July, 2016 (*P* < 0.001)

For patients from January, 2014 to December, 2018, by the end of follow-up, 199 of the 496 patients had relapsed. The follow-up time was 0.89 to 62.33 months. For patients from January, 2014 to July, 2018, by the end of follow-up, 175 of the 422 patients had relapsed. The 1-year relapse-free survival rate was significantly lower in the GLR > 27.5 group (67.7%) than GLR ≤ 27.5 group (84.3%) during follow-up (*P* < 0.001). For patients from January, 2014 to July, 2016, by the end of follow-up, 89 of the 176 patients had relapsed, the median-free survival was 45.77 months. The 1- and 3-year recurrence-free survival rates in the GLR ≤ 27.5 group were 90.9% and 67.0%, respectively; the 1- and 3-year recurrence-free survival rates in the GLR > 27.5 group were 63.2% and 43.7%, respectively (*P* < 0.001). Kaplan–Meier survival curves for the threshold of GLR were shown in Fig. 2.

## Discussion

Our results showed that elevated GLR has a positive correlation with the recurrence of HCC patients with staging I–II after adjustment of age, sex, smoking, operation time, lymphovascular invasion, allogeneic blood, HB, AST, ALP, HBsAg, tumor number, tumor size, AFP. To the best of our knowledge, we were the first to discover a nonlinear relationship between GLR and the risk of recurrence in HCC patients at stage I–II, with a cutoff point of 27.5. The nonlinear relationship was as follows: the hazard ratio (95%CI) of incident recurrence was 1.041 (1.014–1.068) when the GLR was less than 27.5 and 1.001 (1.000–1.003) when the GLR was beyond 27.5. We identify that GLR ≥ 27.5 group had a lower RFS rate than GLR < 27.5 group. Moreover, according to the subgroup analysis, the association between GLR and incident recurrence of HCC patients with staging I–II was modified by HBsAg and AFP.

Inflammatory responses played a vital role in all stages of cancer development and progression [15–17]. In the early stages of cancers, inflammatory cell recruitment created a favorable microenvironment for tumor growth and facilitated the formation of new blood vessel formation [18, 19]. Intrahepatic GGT was chiefly found on the surface of the cell and played a key role in glutathione metabolism [20]. Furthermore, previous studies have shown that increased preoperative increased GGT values were a positive relationship with a poor prognosis of hepatocellular carcinoma [21–23]. GGT had a pro-oxidant effect and catalyzed the generation of reactive oxygen species (ROS), ROS played a proinflammatory function in the NF-κB signaling pathway [22, 24, 25]. Similarly, lymphocytes inhibited tumor proliferation and migration in the human immune system, and increased lymphocyte count predicted a favorable prognosis in patients with various solid tumors [26–28].

In recent years, the prediction model of prognosis of the HCC combined with different inflammation factors has always been a hot issue [9, 10, 29, 30]. Previous studies have examined GLR's prognostic ability in HCC [12, 14], intrahepatic cholangiocarcinoma [31] and nonfunctional pancreatic neuroendocrine tumors [25]. These studies revealed that increased GLR values had a positive correlation with poor long-term outcomes, which matched our findings. However, building a reliable model required us to understand the true relationship between each predictor and the prognosis of HCC, where a non-linear relationship was important. In this study, we found that the relationship between GLR and the recurrence of HCC with staging I–II was nonlinear. This would help us build models in the future.

Subsequently, by subgroup analysis, the association between GLR and the recurrence of HCC with staging I–II was modified by HBsAg and AFP. In HBsAg-negative patients, our study found a significant positive relationship, but this relationship was closely significant (*P* = 0.0589) in HBsAg- positive patients. The previous study found that in HCC patients with AFP-negative after radical resection, GLR had fair accuracy in predicting the early-recurrence, and our findings persisted [13].

Noticeably, to our knowledge, the nonlinear relationship between GLR and the recurrence in HCC with staging I–II was first found. This study, however, has some limitations. First, this study was retrospective and included patients from a single-center hospital. Second, only patients who underwent the first resection and with staging I–II were included in this study. Furthermore, the results of the study were for the Chinese population and may not be applicable to people in other countries. Due to these limitations, multicenter and large-scale studies were warranted to validate our finds.

In conclusion, the increased GLR was independently associated with the recurrence of HCC patients with staging I–II. Furthermore, the relationship between increased GLR and developing recurrence was positive, no-linear and modified by HBsAg and AFP.

## Data Availability

The data that support the findings of this study are available on request from the corresponding author: Jianjun Yang, Department of Anesthesiology, Pain and Perioperative Medicine, The First Affiliated Hospital of Zhengzhou University, Zhengzhou, 450000, China.
